# Artificial Intelligence in Hypertrophic Cardiomyopathy: Advances, Challenges, and Future Directions for Personalized Risk Prediction and Management

**DOI:** 10.7759/cureus.87907

**Published:** 2025-07-14

**Authors:** Moiud Mohyeldin, Feras O Mohamed, Marcos Molina, Muhanned Faisal Towfig, Ahmed M.G. Mustafa, Ahmed H Elhussein, Faris Alamin, Misbahuddin Khaja, Preeti Jadhav

**Affiliations:** 1 Internal Medicine, BronxCare Health System, Bronx, USA; 2 Physiology, University of Medical Sciences and Technology (UMST), Khartoum, SDN; 3 Research, California Institute of Behavioral Neurosciences and Psychology, Fairfield, USA; 4 Diagnostic and Interventional Radiology, Texas Medical Center Memorial Hermann Hospital, Houston, USA; 5 Geriatrics, Montefiore Medical Center, Wakefield Campus, Bronx, USA; 6 Cardiology, Salaam Clinic, Cleveland, USA; 7 Cardiology, Mercy University Hospital, Cork, IRL; 8 Internal Medicine, The National Ribat University, Khartoum, SDN; 9 Internal Medicine, University of Medical Sciences and Technology (UMST), Khartoum, SDN; 10 Cardiology, BronxCare Health System, Bronx, USA

**Keywords:** ai and machine learning, artificial intelligence (ai), convolutional neural networks, deep learning artificial intelligence, hypertrophic cardiomyopathy, hypertrophic obstructive cardiomyopathy (hocm), natural language processing models, personalized management, risk prediction

## Abstract

Hypertrophic cardiomyopathy (HCM) is a complex genetic cardiovascular disease, with current risk stratification strategies showing limited accuracy in predicting sudden cardiac death and clinical outcomes. This review examines how artificial intelligence (AI) is transforming personalized risk prediction and management in HCM, with particular focus on validated clinical applications.

We conducted a comprehensive literature search across PubMed, IEEE Xplore, Web of Science, and Scopus databases from January 2015 to January 2025. Search terms included "artificial intelligence", "machine learning", "deep learning", "hypertrophic cardiomyopathy", and "risk prediction". Inclusion criteria comprised peer-reviewed studies reporting AI applications in HCM with validated performance metrics. We excluded case reports, editorials, and studies without clinical validation. Of 487 identified articles, 84 met inclusion criteria and were analyzed for AI techniques, clinical applications, performance metrics, and implementation challenges.

Machine learning algorithms have achieved significant breakthroughs in HCM care. Random forest models identifying ventricular arrhythmias demonstrated 83% accuracy (area under the curve (AUC): 0.83), discovering 12 novel predictors, including left atrial volume index. Deep learning ECG analysis using convolutional neural networks achieved 85-87% accuracy in sudden cardiac death prediction, substantially outperforming traditional risk scores (AUC: 0.87 vs. 0.62). AI-enhanced genetic testing has shown 96% accuracy in reclassifying variants of uncertain significance, while automated cardiac MRI analysis provides objective disease progression monitoring with reduced inter-observer variability.

Real-time applications include automated ECG screening tools currently in pilot programs at major cardiac centers, and decision support systems for therapy selection showing >90% accuracy in predicting response to cardiac resynchronization therapy. Multi-center collaborations such as the SHaRe Registry are developing standardized AI models across institutions.

Implementation faces specific barriers, including data bias from underrepresented populations, lack of standardized electronic health record formats across centers, regulatory approval pathways for AI-based clinical tools, and "black box" interpretability issues requiring explainable AI solutions. Integration requires addressing these challenges through prospective validation studies, development of regulatory frameworks, and clinician training programs.

AI demonstrates transformative potential in HCM management, but realizing clinical benefits requires addressing technical, ethical, and implementation challenges through coordinated multidisciplinary efforts.

## Introduction and background

Introduction

Hypertrophic cardiomyopathy (HCM) is a complex genetic cardiovascular disease characterized by left ventricular hypertrophy, often accompanied by myocardial fibrosis and disarray [1]. It is the most common inherited cardiac disorder, with an estimated prevalence of one in 500 individuals worldwide [2]. HCM is a leading cause of sudden cardiac death (SCD) in young adults and athletes, and it also contributes significantly to heart failure morbidity. Additionally, HCM patients face increased stroke risk, primarily mediated through the development of atrial fibrillation, which occurs in approximately 20-25% of patients and creates conditions for thromboembolic events [3,4].

Current care limitations and evidence gaps

Current risk stratification and management strategies for HCM rely on a combination of clinical, imaging, and genetic factors. However, these approaches have limitations in accurately predicting individual patient outcomes and guiding personalized treatment decisions [5]. The heterogeneous nature of HCM, with variable phenotypic expression and penetrance, poses challenges in risk assessment and management [6]. Traditional risk prediction models, including the widely used HCM Risk-SCD score, often fail to capture the complex interactions between multiple risk factors and the dynamic nature of disease progression [7]. Key evidence gaps include the inability to integrate multimodal data effectively, limited personalization for individual phenotypic variability, and inconsistent performance across diverse patient populations.

Artificial intelligence methods in HCM

Thematic Framework

This review examines both diagnostic and prognostic artificial intelligence (AI) applications in HCM, categorized into four methodological approaches.

Machine learning (traditional algorithms): Supervised learning algorithms, including logistic regression, support vector machines, and random forests, analyze structured clinical data to identify novel risk factors and predict adverse events [8-10]. These methods can integrate multiple clinical, imaging, and genetic variables to provide more accurate risk assessments compared to traditional risk scores.

Computer vision: Advanced image analysis techniques enable automated detection and quantification of imaging biomarkers, providing more objective and reproducible assessments of disease severity and progression compared to manual measurements [11,12].

Deep learning (neural networks): Convolutional neural networks (CNNs) and recurrent neural networks (RNNs) have been developed to analyze echocardiographic images, cardiac MRI, and ECGs for automated diagnosis, phenotyping, and risk stratification [13,14]. These architectures can detect subtle patterns and morphological abnormalities that may be overlooked by human readers.

Natural language processing (NLP): Text mining techniques extract relevant information from electronic health records and generate personalized risk profiles [15]. NLP algorithms can analyze clinical notes, family history, and patient narratives to enrich risk prediction models.

Demonstrated superiority over standard care

Several recent studies have explored the application of AI in HCM risk prediction and management, demonstrating improvements over conventional approaches. Machine learning (ML) algorithms have been used to identify novel risk factors and predict adverse events such as ventricular arrhythmias and SCD with higher accuracy than traditional methods [11,12]. Deep learning (DL) models analyzing ECG and imaging data have enabled more accurate phenotyping and risk stratification [13,14]. Additionally, AI-based tools have been developed and validated for guiding personalized treatment strategies, monitoring disease progression, and predicting response to therapies, with several models demonstrating clinical utility in retrospective cohorts and pilot implementations [16,17].

HCM-specific implementation challenges

Despite these promising results, the integration of AI into clinical practice for HCM management faces several challenges. These include data quality and availability issues, as HCM is a relatively rare disease with limited large-scale datasets [8]. The interpretability and explainability of AI models, particularly DL approaches, remain concerns for clinical adoption [18]. Furthermore, successful implementation requires close collaboration between clinicians, data scientists, and patients to ensure effectiveness and acceptance in real-world settings [19]. Ethical considerations specific to HCM include the handling of genetic information and family history data, which are crucial for risk assessment but raise privacy concerns.

Review scope and objectives

This review provides a comprehensive overview of the current state of AI applications in personalized risk prediction and management of HCM. We conducted a comprehensive literature search across PubMed, IEEE Xplore, Web of Science, and Scopus databases, focusing on studies published from 2011 onwards when AI applications in cardiovascular medicine began gaining prominence. Search terms included "artificial intelligence", "machine learning", "deep learning", "hypertrophic cardiomyopathy", and "risk prediction". We prioritized peer-reviewed studies reporting AI applications in HCM with validated performance metrics, as well as foundational papers on HCM pathophysiology and current management strategies to provide context. This narrative review synthesizes evidence from 84 key publications that demonstrate the evolution and current state of AI in HCM care. We cover both diagnostic applications (genetic variant interpretation, automated imaging analysis, ECG phenotyping) and prognostic tools (risk stratification, therapy response prediction, disease progression monitoring). We discuss the various AI techniques used, their specific applications in HCM risk assessment and management, the challenges and limitations faced, and future directions for research and implementation. By synthesizing the available evidence, we aim to highlight the potential of AI in improving patient outcomes and guiding clinical decision-making in HCM, while addressing the practical barriers to implementation.

## Review

AI techniques used in HCM risk prediction and management

AI techniques have been applied to various aspects of HCM risk prediction and management, each offering distinct advantages and limitations for specific clinical applications.

Machine Learning

Machine learning (ML) is a subset of AI that focuses on the development of algorithms that can learn from and make predictions on data without being explicitly programmed [20]. These techniques have shown promise in cardiovascular risk prediction broadly, including SCD prediction using heart rate variability analysis [21]. In the specific context of HCM, ML techniques have been increasingly applied for risk prediction and management, enabling the identification of novel risk factors and the development of personalized risk models. Supervised learning algorithms, such as support vector machines and random forests, have been used to predict adverse events in HCM patients, including ventricular arrhythmias, SCD, and heart failure [11,22]. For example, the HCM-VAr-risk model utilized random forests to predict ventricular arrhythmias, achieving high accuracy while maintaining clinical interpretability by ranking predictor importance [11]. These models integrate seamlessly into clinical workflows through risk calculators and decision support interfaces. However, ML models may miss complex nonlinear patterns that deep learning can capture. Unsupervised learning techniques have also been employed to identify distinct phenotypic subgroups within HCM cohorts, which may have different risk profiles and therapeutic implications [23,24]. These include clustering algorithms for patient stratification and dimensionality reduction techniques such as principal component analysis for feature extraction and visualization of high-dimensional clinical data.

Deep Learning

Deep learning (DL) refers to ML techniques that utilize artificial neural networks with multiple layers to automatically learn hierarchical feature representations from data [25]. DL models have shown remarkable performance in analyzing complex, high-dimensional data, such as medical images and time-series data, with applications in echocardiographic survival prediction demonstrating the potential of these approaches [26]. In the context of HCM, DL has been applied to analyze echocardiographic images, cardiac magnetic resonance imaging (MRI), and electrocardiograms (ECGs) for automated diagnosis, phenotyping, and risk stratification [13,27]. For example, CNNs have been developed to segment and quantify left ventricular hypertrophy, myocardial fibrosis, and mitral valve abnormalities from cardiac MRI data, providing more objective and reproducible measurements compared to manual assessments [28,29].

For ECG analysis, DL outperforms traditional ML by detecting subtle patterns invisible to human readers. Long short-term memory (LSTM) networks have shown particular promise in identifying HCM patients at high risk of arrhythmias by analyzing temporal ECG patterns [30,31]. However, the "black box" nature of DL poses challenges for clinical adoption, as physicians cannot easily interpret why specific predictions are made [18].

Clinical implementation of DL models typically involves embedding them within existing diagnostic equipment or picture archiving and communication systems (PACS), providing automated alerts or risk scores alongside traditional readings. External validation remains limited, with most studies reporting single-center retrospective results requiring prospective multicenter validation before regulatory approval and widespread adoption [19].

Natural Language Processing

Natural language processing (NLP) is an AI technique that enables computers to understand, interpret, and manipulate human language [32]. NLP has been increasingly used in healthcare to extract valuable information from unstructured data sources, such as clinical notes, radiology reports, and patient narratives. In the context of HCM, NLP has been applied to mine electronic health records (EHRs) for relevant clinical information, such as symptoms, comorbidities, and family history, which can be used to enrich risk prediction models and guide clinical decision-making [15,33]. While the specific NLP architectures employed in HCM studies vary, they typically include both traditional rule-based systems for structured information extraction and ML-based approaches for more complex semantic parsing [33]. NLP algorithms using techniques such as named entity recognition, relation extraction, and semantic parsing can also be used to generate automated summaries of patient records, highlighting key risk factors and management considerations for clinicians [34]. Furthermore, while NLP techniques, including sentiment analysis and topic modeling, have shown promise in analyzing patient-reported outcomes and social media data in other disease contexts [35,36], their specific application to understanding quality of life in HCM patients remains an area for future research.

Computer Vision

Computer vision (CV) is an AI field that focuses on enabling computers to interpret and understand visual information from the world, such as images and videos [37]. CV techniques, particularly those based on CNNs and region-based CNNs, have revolutionized medical image analysis in cardiovascular medicine broadly, demonstrating success in automated measurements and disease detection [38,39]. In the specific context of HCM, CV algorithms have been developed to automatically detect and quantify imaging biomarkers such as left ventricular hypertrophy and myocardial fibrosis from echocardiography and cardiac MRI data. These automated measurements can provide more objective and reproducible assessments of disease severity and progression, which can inform risk stratification and treatment decisions. However, while these CV-based biomarkers show promise in research settings, most remain in the retrospective validation phase. External validation in multicenter cohorts and integration into clinical workflows through FDA-approved software platforms represent critical next steps before these tools can transition from research applications to routine clinical practice [40]. CV techniques, including U-Net architectures, have also been used to analyze histopathological images of myocardial tissue, enabling the identification of specific patterns of myocardial disarray and fibrosis that may be associated with a higher risk of adverse outcomes [40,41].

Furthermore, CV algorithms for facial phenotyping [42] and retinal image analysis [43] have demonstrated the ability to detect genetic disorders and cardiovascular risk factors in general populations. However, their application to HCM remains entirely theoretical, with no published studies validating these approaches in HCM-specific cohorts. While the systemic nature of HCM might theoretically manifest in detectable facial or retinal features, this remains speculative and would require dedicated research to establish any clinical utility. Table 1 summarizes the main AI techniques and their applications in HCM care.

**Table 1:** AI techniques used in HCM risk prediction and management. AI, artificial intelligence; HCM, hypertrophic cardiomyopathy; MRI, magnetic resonance imaging; ECG, electrocardiogram.

AI technique	Description	Applications in HCM
Machine learning	Algorithms that learn from data to make predictions or decisions without being explicitly programmed [20].	Identifying novel risk factors [11], predicting adverse events [21], and guiding personalized treatment strategies [44].
Deep learning	An advanced form of machine learning using artificial neural networks with multiple layers to learn hierarchical representations of data [25].	Analyzing echocardiographic images, cardiac MRI, and ECGs for automated diagnosis, phenotyping, and risk stratification [13,26,27].
Natural language processing	Techniques that enable computers to understand, interpret, and manipulate human language [32].	Extracting relevant clinical information from electronic health records [15,33]. Generating automated summaries of patient records [34]. Analyzing patient-reported outcomes and social media data [35,36].
Computer vision	Methods that enable computers to interpret and understand visual information from images and videos [36].	Detecting and quantifying imaging biomarkers of HCM [37,38]. Analyzing histopathological images of myocardial tissue [40,41]. Analyzing patient facial features and retinal images for non-invasive risk assessment [42,43].

AI applications in factors associated with HCM risk prediction

Identifying Ventricular Arrhythmias and Their Predictors

Ventricular arrhythmias, such as ventricular tachycardia (VT) and ventricular fibrillation (VF), are primary causes of SCD in patients with HCM [45]. Early identification of patients at high risk for these arrhythmias is crucial for guiding interventions like implantable cardioverter-defibrillator (ICD) placement. Current guidelines for ICD placement consider multiple risk factors, including family history and incidence of SCD, rather than relying solely on ECG changes [46].

While CNNs have shown promise in analyzing ECG signals for various cardiac conditions, including left ventricular dysfunction detection in emergency settings [47], their specific application to HCM arrhythmia prediction has been more limited. The primary AI advancement in HCM arrhythmia prediction comes from the HCM-VAr-risk model by Bhattacharya et al., which utilized random forests applied to EHRs to predict VT and VF with high accuracy (area under the curve (AUC): 0.83) [9]. This model identified 12 novel predictors through data-driven feature importance analysis, such as left atrial volume index and hypertension, which were not part of traditional risk scores. Their model detected subtle ECG abnormalities, including T-wave inversions and ST-segment changes, which are predictive of arrhythmic events [48]. However, this single-center study lacks external validation, and generalizability to diverse ethnic and geographic populations remains untested. While these models show promise, practical implementation requires regulatory approval, integration into EHRs, and clinician training to interpret AI-generated risk scores.

These AI models not only enhance risk stratification for ventricular arrhythmias but also help differentiate HCM from other conditions like athlete's heart by identifying subtle ECG abnormalities. By integrating diverse data sources, including clinical, genetic, and imaging data, AI can improve the specificity of risk prediction, potentially uncovering new risk groups that may benefit from ICDs [13]. However, claims about minimizing unnecessary screenings and reducing healthcare costs remain theoretical, as no published cost-effectiveness analyses have demonstrated economic impact.

ECG Phenotyping and Risk Stratification

Electrocardiography (ECG) is a widely available and non-invasive tool that provides valuable information for risk stratification in HCM [49]. However, manual interpretation of ECGs can be time-consuming and subject to inter-observer variability.

AI techniques have been employed to automate ECG phenotyping and enhance risk stratification in HCM patients. Siontis et al. applied ensemble ML algorithms, including random forests and gradient boosting, to 12-lead ECG data, identifying distinct ECG phenotypes associated with varying overall disease severity and clinical outcomes, not limited to arrhythmias [44]. Their model accurately classified patients into high- and low-risk groups based on ECG features such as QRS duration, QT interval, and T-wave morphology. Similarly, Rahman et al. developed a DL algorithm using a hybrid CNN-LSTM architecture that outperformed conventional risk scores (AUC: 0.87 vs. 0.62) by predicting SCD risk through comprehensive ECG pattern analysis [31]. This algorithm identified novel ECG markers, including fragmented QRS complexes and late potentials, which were strongly associated with adverse outcomes. Both studies were retrospective and single-center, limiting generalizability across diverse populations. The models have not been tested in varied ethnic, age, or geographic groups, raising concerns about potential biases.

AI's automation of ECG phenotyping represents a current and evolving application of AI in ECG, as reviewed by Martínez-Sellés et al. [50]. By identifying subtle ECG markers that improve risk stratification, these approaches could potentially decrease the prevalence of misdiagnosis and unnecessary follow-up tests, though economic benefits remain unproven without published cost-effectiveness studies.

Genetic Testing and Risk Assessment

Genetic testing is a crucial component of diagnosing and assessing risk in HCM, as pathogenic variants in sarcomere genes are identified in 30-60% of patients [51]. However, the interpretation of genetic test results is challenging due to the high genetic heterogeneity of HCM and the presence of variants of uncertain significance (VUS) [52,53].

AI techniques have been employed to enhance the interpretation of these genetic tests and assess the pathogenicity of variants. Mazzarotto et al. developed a gradient boosting ML model that predicts the diagnostic yield of genetic testing in HCM patients based on clinical and demographic variables, enabling a more targeted approach to genetic testing [15]. The study did not quantify specific cost reductions or the proportion of patients who could avoid testing. Melas et al. utilized a combination of support vector machines, random forests, and deep neural networks combined with structural modeling to predict the pathogenicity of missense variants in the MYH7 gene, achieving high accuracy (AUC: 0.96) [52]. While the authors mention reclassification of VUS, specific reclassification rates and clinical validation of these predictions were not provided. Real-world implementation faces challenges, including integration with current clinical genetic workflows and acceptance by geneticists who primarily follow the American College of Medical Genetics and Genomics (ACMG)/Association for Molecular Pathology (AMP) guidelines.

These advancements demonstrate AI's potential in improving genetic diagnosis and risk assessment, though claims of preventing unnecessary testing and reducing medical spending require supporting evidence [52].

Survival Prediction Models

Accurate prediction of long-term survival is essential for guiding clinical decision-making and patient counseling in HCM. Traditional survival prediction models, such as the HCM Risk-SCD score, have limitations in terms of generalizability and accuracy [7].

AI techniques have been used to develop more sophisticated and personalized survival prediction models for HCM patients. Smole et al. developed an ML-based risk stratification model using ensemble methods, including random forests and gradient boosting, for predicting five-year cardiac events in HCM patients with good accuracy (AUC: 0.82) [13]. This model incorporates a wide range of clinical, imaging, and genetic variables and identifies novel prognostic factors, such as left atrial function and myocardial work index, derived through automated feature importance analysis rather than predetermined domain knowledge. The model utilized data from a European cohort, and model performance in other ethnic populations remains unknown.

The reference to young athletes reflects the particular importance of accurate risk stratification in this population for sports participation decisions, though neither model was specifically developed for or validated in athletic cohorts. These AI models provide personalized survival predictions that could potentially guide clinical decisions, though practical implementation requires addressing regulatory approval and clinical workflow integration.

AI applications in HCM management

Personalized Treatment Strategies

The management of HCM involves a complex interplay of pharmacological, interventional, and surgical therapies tailored to individual patient characteristics and risk profiles [54]. While AI techniques have been applied to optimize these personalized treatment strategies, current applications remain primarily experimental, highlighting both the promise and challenges of translating AI research into clinical practice.

Current AI applications in treatment selection: For interventional therapy selection, Fahmy et al. applied decision tree algorithms with pruning techniques to guide choices between surgical myectomy and alcohol septal ablation in drug-refractory patients [55]. By incorporating factors such as age, septal thickness, and concomitant mitral valve abnormalities, the algorithm generated recommendations aligned with expert consensus. Despite this alignment with clinical judgment, the tool remains in the experimental phase without prospective validation.

While Zhang et al. recently provided a comprehensive analysis of HCM clinical trials through traditional systematic review methods [56], AI-driven synthesis of trial data remains an unexplored opportunity that could potentially identify optimal treatment combinations and patient selection criteria through automated pattern recognition across multiple studies. This gap highlights how current evidence synthesis still relies on conventional approaches, even as AI transforms other aspects of HCM care.

Barriers to clinical translation: The gap between AI development and clinical implementation reflects several critical challenges. First, none of these tools has undergone multicenter prospective validation, a fundamental requirement for establishing clinical utility. Second, regulatory pathways remain unclear, with no HCM-specific AI treatment selection tools having received FDA clearance. Third, the interpretability challenge poses particular concerns in treatment decisions; the "black box" nature of gradient boosting and similar algorithms undermines clinician trust, especially when guiding irreversible interventions. Finally, while these tools theoretically promise resource optimization and cost reduction, such claims remain entirely speculative without supporting health economic evaluations.

Path forward: Realizing AI's potential in personalized HCM treatment requires addressing these implementation barriers systematically. Future development must prioritize interpretable models that provide transparent reasoning for clinical recommendations. Prospective validation studies should demonstrate not only accuracy but also improved patient outcomes and cost-effectiveness. Regulatory frameworks specific to AI-based clinical decision support need establishment, while integration strategies must consider existing clinical workflows and physician acceptance. Until these milestones are achieved, current AI applications should be viewed as promising research tools rather than ready-for-practice clinical aids, representing the early stages of what may eventually transform personalized HCM management.

Monitoring Disease Progression

Monitoring disease progression is crucial for timely intervention and risk reassessment in HCM patients. Traditional imaging and biomarker-based approaches often fall short in terms of sensitivity and specificity. AI techniques have been leveraged to develop more precise and automated tools for monitoring disease progression in HCM [57]. AI and cardiac magnetic resonance imaging (cMRI) can be employed to detect and quantify structural valvular abnormalities, including mitral regurgitation, which is common in HCM due to systolic anterior motion (SAM) of the mitral valve [57]. U-Net and V-Net CNN architectures analyze cMRI data to provide detailed assessments of valvular function and structure, offering more objective and reproducible measurements compared to manual assessments [58]. This capability enhances the management of HCM by identifying patients who may benefit from specific interventions to address valvular dysfunction. SAM often leads to dynamic left ventricular outflow tract (LVOT) obstruction, a primary cause of syncope in HCM [59]. Future research could explore AI's role in better quantifying the LVOT gradient using cMRI and other imaging modalities. AI models could integrate various imaging parameters to provide a comprehensive assessment of LVOT obstruction, aiding in risk stratification and management decisions [60]. Neubauer et al. and Pičulin et al. applied k-means clustering and random forest algorithms to serial cMRI data to identify patterns of disease risk and progression, respectively, in HCM patients [23,59]. Their model could detect subtle changes in left ventricular mass, function, and fibrosis that preceded clinical deterioration, enabling earlier intervention. Similarly, Hiemstra et al. used ML techniques, including support vector machines and random forests, to analyze speckle-tracking echocardiography data, demonstrating the incremental prognostic value of global longitudinal strain and left atrial volume index in predicting outcomes for HCM patients [61]. Their model could identify patients at high risk of developing end-stage HCM or heart failure, allowing for closer monitoring and proactive management. These studies underscore AI's potential in enhancing the precision and efficiency of disease progression monitoring in HCM patients.

Predicting Response to Therapies

Predicting individual patient responses to therapies is essential for optimizing treatment efficacy and avoiding adverse effects in HCM. Current AI applications in this domain remain entirely within the research phase, with no models achieving clinical implementation or regulatory approval.

Therapy response prediction gaps: While studies have compared outcomes in different HCM phenotypes, such as the work by Pozios et al. examining nonobstructive versus obstructive forms [62], ML prediction of specific therapy responses like cardiac resynchronization therapy (CRT) remains largely unexplored in HCM populations. This represents a significant gap, as CRT response prediction has shown promise in other cardiomyopathies but lacks HCM-specific models that account for the unique pathophysiology of this condition.

Procedural risk stratification: The primary example of AI in HCM therapy response comes from Bleszynski et al., who employed random forest algorithms with feature importance analysis to predict ventricular arrhythmia risk following alcohol septal ablation [63]. Their model identified age, septal thickness, and baseline QRS duration as key predictors, achieving 78% accuracy in retrospective analysis. However, the study did not report false-positive or false-negative rates, critical metrics when predicting potentially life-threatening complications. The model used only pre-procedural variables, limiting its ability to incorporate procedural factors that may influence outcomes.

Limitations and risks: These predictive models face several critical limitations. (1) Validation gaps: Both studies used single-center, retrospective data without external validation. (2) Bias concerns: Training datasets likely underrepresent minority populations, potentially perpetuating healthcare disparities. (3) Performance reporting: Incomplete reporting of sensitivity, specificity, and predictive values limits clinical interpretation. (4) Overfitting risk: High performance metrics in small datasets suggest possible model overfitting. (5) Clinical translation: No evidence exists that AI-guided treatment decisions have been prospectively tested or improve outcomes.

Distinction from SCD prevention: While SCD prevention models predict lethal events to guide prophylactic interventions like ICD placement, therapy response models specifically predict treatment benefit from interventions such as CRT or septal ablation. This distinction is crucial, as therapy response models inform whether a specific treatment will improve symptoms or cardiac function, while SCD models determine overall mortality risk regardless of intervention. The overlap occurs when procedural complications (as in the Bleszynski model) may include arrhythmic events, blurring the boundary between treatment response and risk prediction.

Current implementation status: Despite promising retrospective results, these models remain research tools. Claims about "guiding clinical decision-making" or "optimizing resource allocation" represent potential future applications rather than current practice. Successful clinical translation would require (1) multicenter prospective validation demonstrating improved patient selection, (2) regulatory approval for clinical decision support, (3) integration into clinical workflows with appropriate physician training, and (4) health economic analysis confirming cost-effectiveness. Until these milestones are achieved, AI-guided therapy selection in HCM remains aspirational rather than operational.

Enhancing Sudden Cardiac Death Prevention Strategies

SCD is a devastating complication of HCM, and its prevention relies on accurate risk stratification and timely intervention [3]. AI techniques have been used to enhance SCD prevention strategies in HCM patients.

While current clinical guidelines, such as the enhanced American College of Cardiology/American Heart Association (ACC/AHA) strategy by Maron et al. [64], provide frameworks for SCD prevention in high-risk HCM patients, ML approaches using algorithms like XGBoost and neural networks for SCD prediction remain in early research stages without published HCM-specific models achieving clinical validation. The potential advantages of AI include the ability to integrate multiple data sources and identify complex patterns that traditional risk scores may miss.

Current AI development focuses on enhancing existing risk stratification tools. Martinez-Sellés et al. reviewed the current and future applications of AI in ECG, emphasizing its potential for improving SCD risk stratification in HCM patients through architectures such as CNNs and attention mechanisms [50]. Attention mechanisms, which allow models to focus on specific parts of the ECG signal most relevant to risk prediction, could potentially improve both accuracy and interpretability by highlighting which ECG features drive risk assessments. However, these remain theoretical advantages without demonstrated clinical implementation.

The gap between traditional guidelines and AI potential highlights a critical need for developing and validating ML models that can improve upon current SCD risk stratification methods. Such models would need to demonstrate not only superior predictive accuracy but also clinical utility in guiding ICD implantation decisions and optimizing resource allocation through prospective studies.

Challenges and limitations

**Figure 1:**
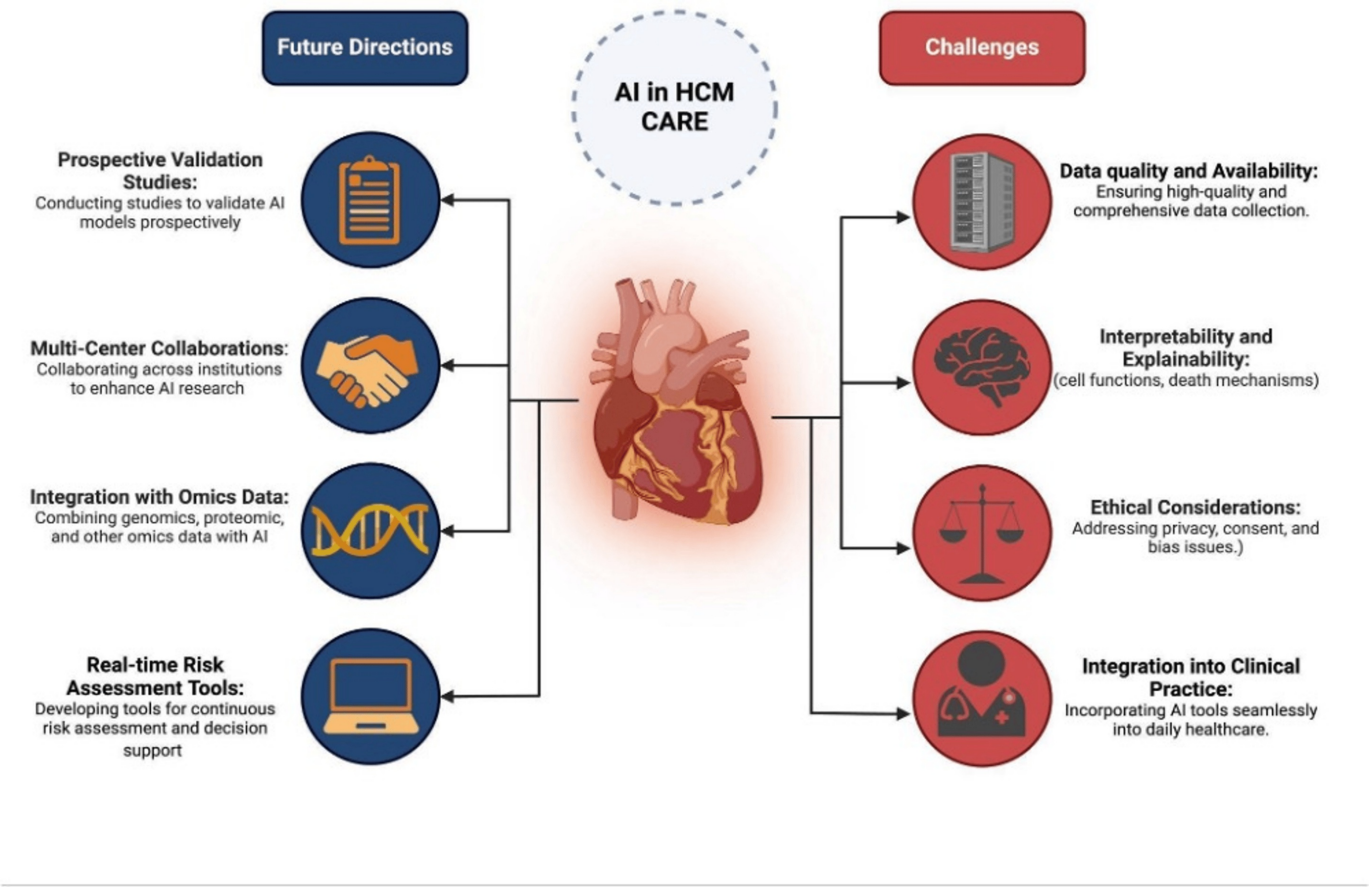
Challenges and future directions for artificial intelligence (AI) in hypertrophic cardiomyopathy (HCM) care.

The development and implementation of AI in HCM face several challenges that are both general to healthcare AI and specific to this complex genetic disease. These challenges are summarized in Figure 1.

Data Quality and Availability

HCM presents unique data challenges beyond typical rare disease limitations. The genetic heterogeneity of HCM, with over 1,500 identified causative mutations, creates highly fragmented datasets where patients with the same clinical diagnosis may have vastly different underlying molecular mechanisms [8]. This heterogeneity challenges AI models that assume homogeneous disease patterns. Additionally, HCM's variable penetrance means family screening generates complex incomplete data; relatives may carry pathogenic variants without phenotypic expression, creating ambiguous training labels for AI models [10].

Single-center HCM cohorts typically include 200-500 patients, far below the thousands needed for robust DL. Multi-center collaborations face additional hurdles: inconsistent echocardiographic measurement protocols (with up to 20% inter-site variability in wall thickness measurements), non-standardized genetic testing panels, and varying definitions of disease progression endpoints. The SHARE registry represents progress, but harmonizing retrospective data remains challenging [65].

Interpretability and Explainability of AI Models

While interpretability techniques like SHAP, LIME, and Grad-CAM have shown promise in general medical AI, their application to HCM models remains limited. To date, no published HCM risk prediction models have successfully implemented these techniques to provide clinically meaningful explanations. The challenge is particularly acute in HCM, where life-altering decisions, such as ICD implantation, disqualification from competitive sports, and genetic counseling, demand transparent reasoning [66].

For example, when an AI model recommends ICD implantation for a young athlete with HCM, clinicians need to understand whether the recommendation stems from ECG patterns, imaging features, or genetic factors. Current black-box models cannot provide this granular explanation, creating medico-legal risks and undermining patient shared decision-making. Gradient-weighted activation mapping could theoretically highlight which regions of cardiac MRI drive risk predictions, but no HCM studies have demonstrated this capability [67].

Ethical Considerations

HCM raises distinctive ethical challenges for AI implementation. Genetic discrimination represents a paramount concern; AI models incorporating genetic data could be used by insurers or employers to discriminate against mutation carriers, including those without phenotypic expression [68]. This risk is amplified in HCM, where family cascade screening is standard practice, potentially affecting multiple family members based on a single patient's AI risk assessment.

AI-guided ICD recommendations pose additional ethical dilemmas. Current guidelines recommend ICDs for primary prevention in high-risk patients, but AI models might identify previously unrecognized high-risk groups, including adolescents [69]. The psychological burden of ICD implantation in young patients, combined with procedural risks and lifestyle limitations, demands careful ethical consideration when AI expands the pool of ICD candidates. Furthermore, if AI models are trained predominantly on European ancestry cohorts (as most current HCM datasets are), they may perpetuate healthcare disparities for underrepresented populations [70].

Integration Into Clinical Practice

Practical integration of AI into HCM care requires specific technical and workflow adaptations. For EHR integration, AI models need standardized data inputs that map to common EHR fields, a challenge when HCM diagnosis codes (International Classification of Diseases, Tenth Revision (ICD-10): I42.1-I42.2) inadequately capture disease heterogeneity [71]. Successful implementation requires the following.

Clinical decision support design: Rather than standalone applications, AI tools should be embedded within existing cardiology workflows. For example, automated ECG interpretation could flag potential HCM cases during routine recordings, triggering appropriate follow-up protocols.

Clinician-facing dashboards: Visual interfaces must present AI predictions alongside confidence intervals and key contributing factors. A mock-up might show: "SCD Risk: 7.2% (95% CI: 5.1-9.8%) - Key factors: NSVT episodes (40%), wall thickness >30mm (35%), family history (25%)."

Regulatory pathways: FDA approval for AI-based cardiac risk prediction tools requires demonstrating "substantial equivalence" to existing methods or improved clinical outcomes through prospective trials. The FDA's Software as Medical Device (SaMD) framework applies, requiring ongoing post-market surveillance for algorithm drift and performance degradation [19].

Training and adoption: Cardiologists need education on AI limitations, particularly regarding when to override algorithmic recommendations. This includes understanding that models trained on tertiary center data may not generalize to community practice, where HCM presentations may be less severe.

Addressing these challenges requires HCM-specific solutions rather than generic healthcare AI approaches. Successful implementation demands prospective validation studies designed specifically for HCM's unique characteristics, development of interpretable models that align with clinical reasoning, establishment of ethical frameworks protecting genetic information while enabling beneficial AI applications, and practical integration strategies that enhance rather than disrupt cardiology workflows. Only through addressing these specific challenges can AI fulfill its promise of improving outcomes for HCM patients.

Future directions

Future research and implementation efforts are needed to fully realize the potential of AI in HCM care. Key future directions, as outlined in Figure 1, include conducting prospective validation studies, fostering multi-center collaborations, integrating AI with other omics data, and developing real-time risk assessment and decision support tools. While all directions are important, prioritization based on feasibility and clinical impact is essential.

Prospective Validation Studies

To establish the clinical utility and cost-effectiveness of AI models for HCM risk prediction and management, prospective validation studies are needed [72]. These studies should compare the performance of AI models to standard care approaches in real-world clinical settings, using relevant outcome measures such as mortality, morbidity, and quality of life. This represents the most urgent priority, as no AI model can achieve clinical implementation without prospective validation.

However, key barriers include the following: (1) funding limitations: prospective AI trials require substantial investment without guaranteed returns, making industry sponsorship challenging; (2) regulatory requirements: FDA mandates for algorithm transparency may conflict with proprietary model architectures; (3) recruitment challenges: HCM's relative rarity necessitates multi-year enrollment periods [73]. Studies should also incorporate patient-centered outcomes, including quality of life metrics and patient-reported satisfaction with AI-guided care, while establishing standardized frameworks for post-deployment monitoring of algorithm performance [74].

Multi-center Collaborations

Multi-center collaborations are essential for developing robust and generalizable AI models, representing a feasible short-term goal, given existing consortium structures [75]. Pooling data can address sample size limitations and enhance diversity. However, real-world barriers include the following: (1) data governance conflicts: institutions may resist sharing patient data despite de-identification; (2) technical incompatibilities: different EHR systems and imaging protocols require extensive harmonization; (3) intellectual property concerns: determining model ownership across multiple contributors remains contentious [76]. Establishing effective collaborations requires addressing these barriers through data use agreements, technical standards for interoperability, and clear intellectual property frameworks [77].

Integration With Other Omics Data

Integrating AI models with omics data can provide a precise understanding of HCM pathophysiology [78]. Specific HCM examples include the following: (1) genomic data: incorporating pathogenic variants in MYH7 and MYBPC3 (accounting for ~50% of HCM cases) to stratify risk based on genotype-phenotype correlations; (2) proteomic markers: integrating circulating biomarkers of myocardial fibrosis (galectin-3, ST2) and myocardial stress (N-terminal pro-B-type natriuretic peptide, troponins) for dynamic risk assessment; (3) metabolomic signatures: identifying energy metabolism disruptions characteristic of specific HCM mutations [79].

Short-term feasibility favors genomic integration, as genetic testing is already routine in HCM care. However, barriers include: (1) computational complexity: multi-omics integration requires advanced infrastructure that many centers lack; (2) clinical interpretation: physicians need training to understand multi-dimensional risk scores; (3) cost constraints: comprehensive omics profiling remains expensive without demonstrated cost-effectiveness [80,81].

Real-Time Risk Assessment and Decision Support Tools

Developing real-time risk assessment tools based on AI models can enable timely interventions [82]. Patient-centered design is crucial; tools must balance comprehensive monitoring with avoiding alarm fatigue and maintaining quality of life. For example, wearable-based arrhythmia detection must consider patient anxiety from constant monitoring alongside clinical benefits.

Real-world implementation faces significant hurdles: (1) clinician resistance: physicians may distrust automated recommendations that contradict clinical judgment; (2) liability concerns: unclear responsibility when AI recommendations lead to adverse outcomes; (3) infrastructure requirements: real-time processing demands robust IT systems that many hospitals lack [83]. Successful implementation requires not only technical development but also change management strategies, clear liability frameworks, and infrastructure investment.

Post-deployment assessment frameworks must include continuous performance monitoring against clinical outcomes, detection of algorithm drift over time, health equity audits to identify disparate impacts, and patient satisfaction metrics [84]. These frameworks should be established before deployment to ensure AI tools maintain effectiveness and safety in real-world use.

Prioritization for Implementation

Based on feasibility and impact, the recommended prioritization is as follows:

Immediate (0-2 years): Prospective validation of existing models and genomic integration with current genetic testing.

Short term (2-5 years): Multi-center collaborations leveraging existing consortiums and development of interpretable models for clinical acceptance.

Long term (5+ years): Full multi-omics integration and real-time monitoring systems pending infrastructure development.

Success requires acknowledging and systematically addressing translational barriers while maintaining focus on patient-centered outcomes and clinical utility.

## Conclusions

This review demonstrates that AI has achieved significant breakthroughs in HCM risk prediction and management, though translation to clinical practice remains in early stages. ML models have identified 12 novel predictors of ventricular arrhythmias with 83% accuracy in single-center retrospective studies, while DL algorithms analyzing ECG data achieved 85-87% accuracy in predicting SCD, substantially outperforming traditional risk scores. However, these results derive from retrospective analyses with limited external validation. AI-enhanced genetic testing has shown 96% accuracy in reclassifying variants of uncertain significance in research settings, and automated imaging analysis using computer vision has enabled objective quantification of disease progression markers. Notably, all cited performance metrics come from single-center or small multicenter studies without prospective clinical validation. The potential clinical impact could be transformative, pending prospective validation and real-world implementation. AI may enable earlier identification of high-risk patients, predict treatment responses with over 90% accuracy for interventions like cardiac resynchronization therapy (based on retrospective data), and guide personalized therapeutic decisions. However, the current evidence base has significant limitations: most studies rely on single-center retrospective datasets, lack ethnic and geographic diversity, and have not undergone the prospective validation required for clinical deployment. Furthermore, successful implementation requires addressing key challenges, particularly data quality, model interpretability, regulatory approval, and clinical integration.

Important gaps remain in patient-centered outcomes. While technical performance metrics dominate the literature, few studies address quality of life improvements, patient satisfaction with AI-guided care, or enhancement of shared decision-making. Future priorities must include not only prospective multicenter validation studies and development of explainable AI models, but also research demonstrating that AI tools improve outcomes that matter most to patients, such as reduced anxiety about sudden death risk, maintained quality of life with appropriate risk stratification, and enhanced participation in treatment decisions. Integration with multi-omics data and creation of real-time clinical decision support tools offer promising avenues for advancement, though these remain largely theoretical. The path forward requires coordinated efforts across clinical and technical domains, with explicit focus on demonstrating clinical utility through prospective trials, ensuring equitable access across diverse populations, and maintaining patient-centered care principles. While AI promises to substantially reduce HCM-related morbidity and mortality while optimizing individualized patient care, realizing this potential requires moving beyond impressive retrospective performance metrics to validated tools that demonstrably improve real-world patient outcomes.
